# Gestational diabetes-combined excess weight gain exacerbates gut microbiota dysbiosis in newborns, associated with reduced abundance of *Clostridium*, *Coriobacteriaceae*, and *Collinsella*

**DOI:** 10.3389/fcimb.2024.1496447

**Published:** 2024-12-13

**Authors:** Yunshan Xiao, Yuan Shi, Yan Ni, Meilan Ni, Yuxin Yang, Xueqin Zhang

**Affiliations:** ^1^ Department of Obstetrics, Women and Children's Hospital, School of Medicine, Xiamen University, Xiamen, China; ^2^ Xiamen Key Laboratory of Basic and Clinical Research on Major Obstetrical Diseases, Women and Children's Hospital, School of Medicine, Xiamen University, Xiamen, China; ^3^ Xiamen Clinical Research Center for Perinatal Medicine, Xiamen Obstetric Quality Management Center, Xiamen, China

**Keywords:** gestational diabetes mellitus, excessive gestational weight gain, newborns, gut microbiota, 16S rRNA

## Abstract

**Background:**

Existing literature indicates that Gestational diabetes mellitus (GDM) and maternal obesity disrupt the normal colonization of the neonatal gut microbiota alone. Still, the combined impact of GDM and excessive gestational weight gain (EGWG) on this process remains under explored. The association between gestational weight gain before/after GDM diagnosis and neonatal gut microbiota characteristics is also unclear.The purpose of this study is to conduct investigation and analysis on the above-mentioned issues, providing a basis for optimizing clinical management plans.

**Methods:**

This study involved 98 mother-infant pairs categorized into GDM and non-GDM groups. The GDM group was further subdivided based on gestational weight gain (GWG) into normal (GDM+NGWG) and excessive (GDM+EGWG) weight gain groups. Neonatal stool samples were collected within 24 hours post-delivery for gut microbiota profiling through 16S rRNA gene sequencing. Statistical analyses explored correlations between total GWG/BMI gain and those before/after GDM diagnosis (t-GWG/GBG; b-GWG/GBG; a-GWG/GBG) with key bacterial taxa.

**Results:**

Notable genus-level changes included enrichment of *Escherichia* and *Klebsiella*, and depletion of *Bacteroides*, *Bifidobacterium*, *Coprococcus*, *Ruminococcus* among GDM-Total and GDM+EGWG groups compared to non-GDM. Further,LEfSe analysis identified 30 differential bacteria taxa between GDM-Total and healthy control groups, which increased to 38 between GDM+EGWG and non-GDM groups, highlighting more pronounced microbial shifts associated with EGWG. *Clostridium* was negatively correlated with t-GWG and newborn birth weight; The *Coriobacteriaceae* showed a negative correlation with t-GWG, t-GBG, and a-GBG. Additionally,*Collinsella* exhibited negative correlations with t-GBG and a-GBG.

**Conclusion:**

This study has identified that the presence of EGWG in GDM mothers further exacerbated neonatal gut microbial perturbations. Total GWG/GBG and those after the diagnosis of GDM were negatively correlated with the abundance of neonatal gut *Clostridium*, *Coriobacteriaceae*, and *Collinsella*. These findings provide new insights for precise prevention and management of GDM.

## Introduction

Gestational diabetes mellitus (GDM) is a condition characterized by glucose tolerance identified during pregnancy, affecting approximately 16.7% of pregnancies globally, according to the International Diabetes Federation (IDF) (Federation). GDM not only increases the risk of cesarean section, dystocia, preterm birth, and postpartum type 2 diabetes in pregnant women but also has complex and persistent adverse effects on their offspring (Xiang, 2023; Wicklow and Retnakaran, 2023). Offspring born to mothers with GDM are significantly increased risk of developing various glucose and lipid metabolism disorders and cardiovascular diseases in childhood or adulthood. Moreover, these conditions may even affect the subsequent generations through epigenetic modification, forming a vicious “intergenerational cycle” (Liu et al., 2023; Perng et al., 2020; Li et al., 2022; Mora-Janiszewska et al., 2022).

The offspring of pregnant women with GDM or excessive gestational weight gain (EGWG) are at increased risk for a range of health issues, including macrosomia, neonatal hypoglycemia, a low Apgar score, and hyperbilirubinemia (Huang et al., 2023).Research has shown that the impact of GDM and EGWG on offspring health can extend beyond birth. A birth cohort study from Hefei, China, reported that GDM and EGWG significantly increased the likelihood of obesity and various metabolic diseases in offspring up to two years after birth (Yin et al., 2023). Similarly, findings from a German birth cohort indicated that GDM and EGWG may disrupt autonomic nervous system (ANS) development, with effects persisting in two-year-old children (Fritsche et al., 2022). These studies highlight the potential long-term risks associated with maternal GDM and EGWG on child health.

These risks may be linked to changes in the gut microbiota characteristics of newborns. Disruptions in gut microbiota can have significant short-term and long-term impacts (Mora-Janiszewska et al., 2022; Wang et al., 2018). Although infant gut microbiota typically stabilizes 2-3 years after birth, its early establishment during the intrauterine or neonatal period has a lasting impact on development (Ray, 2016; Donald and Finlay, 2023).If disrupted during this critical period, infants may face increased health risks later in life.

The characteristics of the neonatal gut microbiota are closely related to the maternal state during pregnancy. It has been reported that GDM and pre-pregnancy obesity can interfere with the establishment of the neonatal gut microbiota (Ren et al., 2023; Guzzardi et al., 2022; Zhu et al., 2022; Zhou and Xiao, 2018; Song et al., 2023). However, the impact of GDM combined with EGWG on the neonatal gut microbiota remains poorly understood. We hypothesize that when pregnant women with GDM experience unsatisfactory weight management leading to EGWG, the characteristics of their offspring’s gut microbiota will differ from those of GDM mothers with normal gestational weight gain (NGWG). To verify this hypothesis, we conducted a study aimed at providing a basis for optimizing the clinical management of GDM pregnancy and implementing targeted, personalized, dynamic, and precise prevention and control measures to reduce adverse perinatal outcomes.

## Materials and methods

### Recruitment of study participants

**Figure 1:**
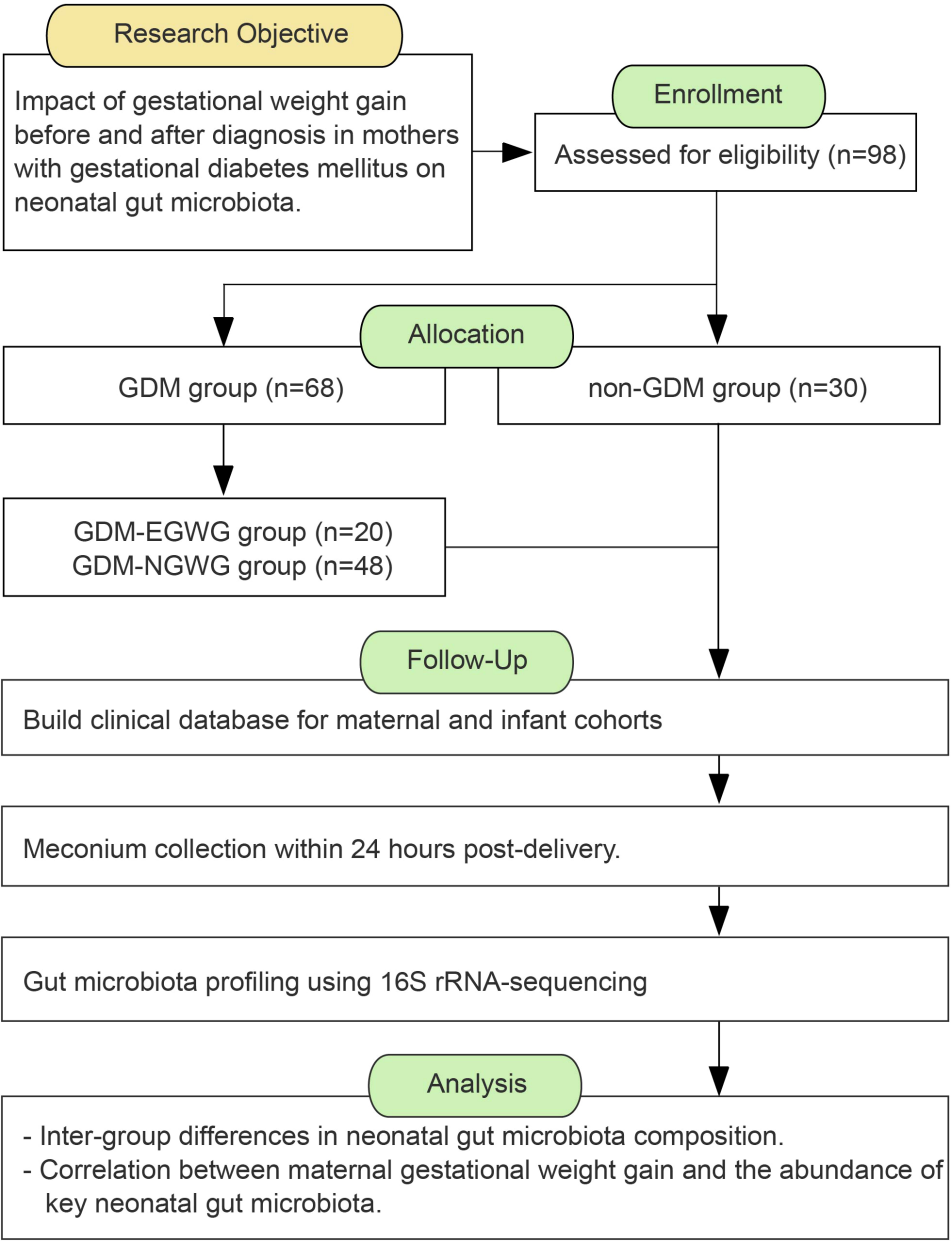
The workflow of study design.

This study is a prospective cohort study.Mothers and infants who were hospitalized for delivery at Women and Children’s Hospital, Xiamen University, from October 2021 to October 2022 were recruited for this study (**Figure 1**). The inclusion criteria were as follows: full-term singleton pregnancy, vaginal delivery; regular prenatal care with complete clinical information; no use of medication affecting glucose metabolism during pregnancy; no antibiotics or probiotics within 2 weeks before delivery; no significant congenital anomalies or chromosomal abnormalities in the neonates. no family history of diabetes, no history of smoking, alcohol, or drug use. The pregnant women participating in this study were between the ages of 22 and 45, had no serious medical or surgical conditions, and were long-term residents of Xiamen City, Fujian Province, having lived there for over 10 years. All participants registered for prenatal care at the Affiliated Women’s and Children’s Hospital of Xiamen University upon confirming pregnancy and received regular check-ups until delivery. Dietary and lifestyle guidance was provided by a consistent medical team according to standard protocols, with all newborns exclusively breastfed. The study received ethical approval from the Ethics Committee of Women and Children’s Hospital, Xiamen University (Ethics Review Number: KT-2021-03-K02). The Clincal Registry number from the China Clinical Trial Registration Center is ChiCTR2100050968. Participants were informed about the study’s objectives and provided their written informed consent. All interactions with participants were conducted in accordance with established guidelines and ethical standards.

Participants were divided into two groups based on their GDM diagnosis and perinatal outcomes: a healthy control group with normal gestational weight gain and both the mothers and their newborns healthy (non-GDM group), and a GDM group (GDM-Total group). The GDM-Total group was further subdivided into two subgroups: those with excessive gestational weight gain (GDM+EGWG group) and those with normal gestational weight gain (GDM+NGWG group).

According to the International Association of Diabetes and Pregnancy Study Groups (IADPS) criteria, OGTT was scheduled between 24 and 28 weeks of gestation (International Association of et al., 2010). For this study, to minimize gestational age variation, OGTT testing was limited to between 25 and 26 weeks of gestation for all participants. GDM diagnosis was based on the criteria established by IADPSG, identifying women with fasting glucose ≥ 5.1 mmol/L, 1-hour glucose ≥ 10.0 mmol/L, or 2-hour glucose ≥ 8.5 mmol/L. The management of GDM in this study involved dietary and exercise interventions, with no use of medications for glycemic control.

### Measurement and calculation of parameters

Pre-pregnancy weight was obtained based on self-reported weight recalled by participants during their first prenatal visit, a method widely accepted in pregnancy studies despite potential recall bias (Kennedy et al., 2023; Li et al., 2021).Height was measured and recorded at the first prenatal visit to ensure accuracy. BMI=Weight/Height^2^(kg/m^2^), total gestational weight gain(t-GWG) = Weight at delivery - Pre-pregnancy weight, total gestational BMI gain(t-GBG)= BMI at delivery - Pre-pregnancy BMI, gestational weight gain before OGTT(b-GWG) = Weight at OGTT - Pre-pregnancy weight, gestational BMI gain before OGTT(b-GBG)= BMI at OGTT - Pre-pregnancy BMI.gestational weight gain after OGTT (a-GWG) = Weight at delivery - Weight at OGTT, gestational BMI gain after OGTT(a-GBG) = BMI at delivery - BMI at OGTT.

The women included in the study had pre-pregnancy BMIs ranging from 18.5 to 24.0 kg/m^2, classified as normal by the Chinese Obesity Working Group (Zhou and Cooperative Meta-Analysis Group of the Working Group on Obesity in, 2002). Normal gestational weight gain (NGWG) was set at 8–14 kg following the “Standard for Monitoring and Evaluating Gestational Weight Gain” (T/CNSS 009–2021) issued by the Chinese Nutrition Society, Any weight gain excedding 14 kg was classified as excessive (EGWG) (Chen et al., 2022). Additionally, we documented total gestational BMI gain (t-GBG). The weight and BMI of pregnant women were recorded during the OGTT. Gestational weight gain and BMI changes before and after the OGTT (b-GWG/b-GBG, a-GWG/a-GBG) were calculated based on weight differences between pre-pregnancy and pre-delivery measurements.

Clinical information, including maternal age, height, pre-pregnancy weight, gravidity, BMI, gestational weight gain, gestational age at delivery, history of antibiotic usage, infant gender, and birth weight, was extracted from the hospital’s electronic medical records.

### Sample collection and processing, DNA extraction, and 16S rRNA gene sequencing

Neonatal meconium was collected within 24 hours post-delivery using sterile techniques and preservation solutions provided in the MGIEasy Stool Sample Collection kit. Over 10 grams of feces were collected per sample, stored in tubes, and immediately frozen at -80°C for subsequent DNA extraction.

Genomic DNA was extracted using the MoBio PowerSoil DNA Isolation Kit according to the manufacturer’s guidelines. The quality and concentration of the extracted DNA were verified through agarose gel electrophoresis. The V3-V4 hypervariable regions of the 16S rRNA gene were amplified using specific primers (341F/806R), facilitating high-resolution microbiota profiling. The amplified DNA was sequenced on the MGISEQ-2000 system using combinatorial Probe-Anchor Synthesis (cPAS) technology. The sequence data have been deposited in the NCBI Sequence Read Archive. (BioProject ID: PRJNA1108428).

#### Microbial sequence data processing

In the microbiome analysis, OTUs (Operational Taxonomic Units) were used for microbial classification. The 16S rRNA gene sequences were analyzed using QIIME software (version 1.8) (Caporaso et al., 2010). No covariates were included in the analysis. To ensure the generation of high-quality clean reads, several quality filtering steps were applied. First, reads with an average Phred quality score below 20 over a 30 bp sliding window were truncated. If reads were reduced to less than 75% of their original length after truncation, they were discarded. Additionally, reads contaminated with adapter sequences or containing ambiguous bases (N bases) were removed. Finally, reads classified as low complexity were excluded from further analysis.

#### Statistical analysis of microbiota data

Statistical analyses were conducted using SPSS version 22.0. Continuous variables were assessed using the Wilcoxon test, while categorical variables were evaluated using the Chi-squared test, with significance set at *P* < 0.05.Alpha diversity indices, including Shannon diversity and Chao index, were analyzed using the Wilcoxon rank-sum test. Beta diversity was explored using unweighted UniFrac distances, which were visualized through principal coordinate analysis (PCoA) to assess microbial community dissimilarities among samples.

#### Taxonomic and functional analysis

Linear discriminant analysis (LDA) integrated with the LEfSe algorithm was employed to identify statistically significant bacterial taxa across various taxonomic levels, from phylum to genus.An LDA score threshold of 4.0 set to determine significance. The metabolic functional profiles of microbial communities were predicted using PICRUSt2.0 within the QIIME2 framework (Douglas et al., 2020).

#### Network analysis

Ecological networks were constructed using the SpiecEasi R package, which employs Sparse Inverse Covariance Estimation to infer ecological associations among microbial genera (Kurtz et al., 2015).

#### Correlation analysis

Spearman’s correlation analysis was utilized to explore the relationships between key gut bacterial taxa and gestational weight gain indicators. Significant correlations were defined by Spearman’s rho values less than -0.3 or greater than 0.3, with an FDR-corrected P value of less than 0.002 (Weiss et al., 2016). Key bacterial taxa, identified by significant differences in relative abundance between the GDM+EGWG and GDM+NGWG groups and an LDA score above 3.0, were analyzed for their associations with total gestational weight gain (t-GWG/t-GBG), as well as weight gain before and after the OGTT (b-GWG/b-GBG, a-GWG/a-GBG).

## Results

### Cohort characteristics

There were no statistically significant differences in maternal age, gestational weeks, parity, pre-pregnancy BMI, or fetal sex between the non-GDM, GDM+NGWG, and GDM+EGWG groups (*P* > 0.05), confirming that the baseline characteristics were similaracross the groups (**Table 1**). However, compared with to the non-GDM group, the birth weight of neonates in the GDM+EGWG group was significantly higher (*P* < 0.05).

**Table 1:** Clinical information of mothers and newborns.

Variable	non-GDM (30)	GDM+NGWG (48)	*P*1	GDM+EGWG (20)	*P*2
Age(years)	32(29,33)	33(30,35)	0.06	32(29,35)	0.41
Primiparous	11(37.7%)	25(52.1%)	0.18	9(45.0%)	0.56
Multigravidas	19(62.3%)	23(47.9%)		11(55.0%)	
ppBMI	21.1(19.8,22.4)	20.9(19.4,22.4)	0.53	21.1(19.4,22.8)	0.91
female	12(40.0%)	21(43.7%)	0.74	7(35.0%)	0.72
Male	18(60.0%)	27(56.3%)		13(65.0%)	
Gestational weeks	39.7(39.3,40.1)	39.4(39.0,40.0)	0.12	39.4(38.8,40.1)	0.37
OGTT FBG(mmol/L)	4.5(4.1,4.6)	4.7(4.5,5.1)	0.001	4.6(4.4,5.0)	0.04
OGTT 1h(mmol/L)	7.7(6.3,8.4)	10.3(9.2,11.0)	0.000	9.8(8.9,10.9)	0.00
OGTT 2h(mmol/L)	6.6(6.0,7.5)	9.1(8.5,9.7)	0.000	9.3(77,9.8)	0.00
Gestational weight gain, kg	10.8(8.4,13.1)	10.1(9.0,11.3)	0.46	15.0(14.2,17.0)	0.00
t-GBG	4.19(3.3,5.1)	4.01(3.5,4.6)	0.35	6.34(5.4,7.3)	0.00
Weight at birth (g)	3306(3097,3422)	3253(2938,3514)	0.94	3451(3213,3652)	0.03

P1: non-GDM and GDM+NGWG; P2: non-GDM and GDM+EGWG;

PpBMI, Pre-pregnancy BMI.

### Overview of microbiota analysis in GDM-related groups

In our study, we compared the gut microbiota characteristics of neonates across different GDM-related groups. Analysis using unweighted UniFrac distances for beta diversity and LEfSe for identifying differential bacteria revealed distinct microbial community structures and significant variations in bacterial composition at both phylum and genus levels. and significant variations in bacterial composition at both phylum and genus levels.

### Alpha and beta diversity

**Figure 2:**
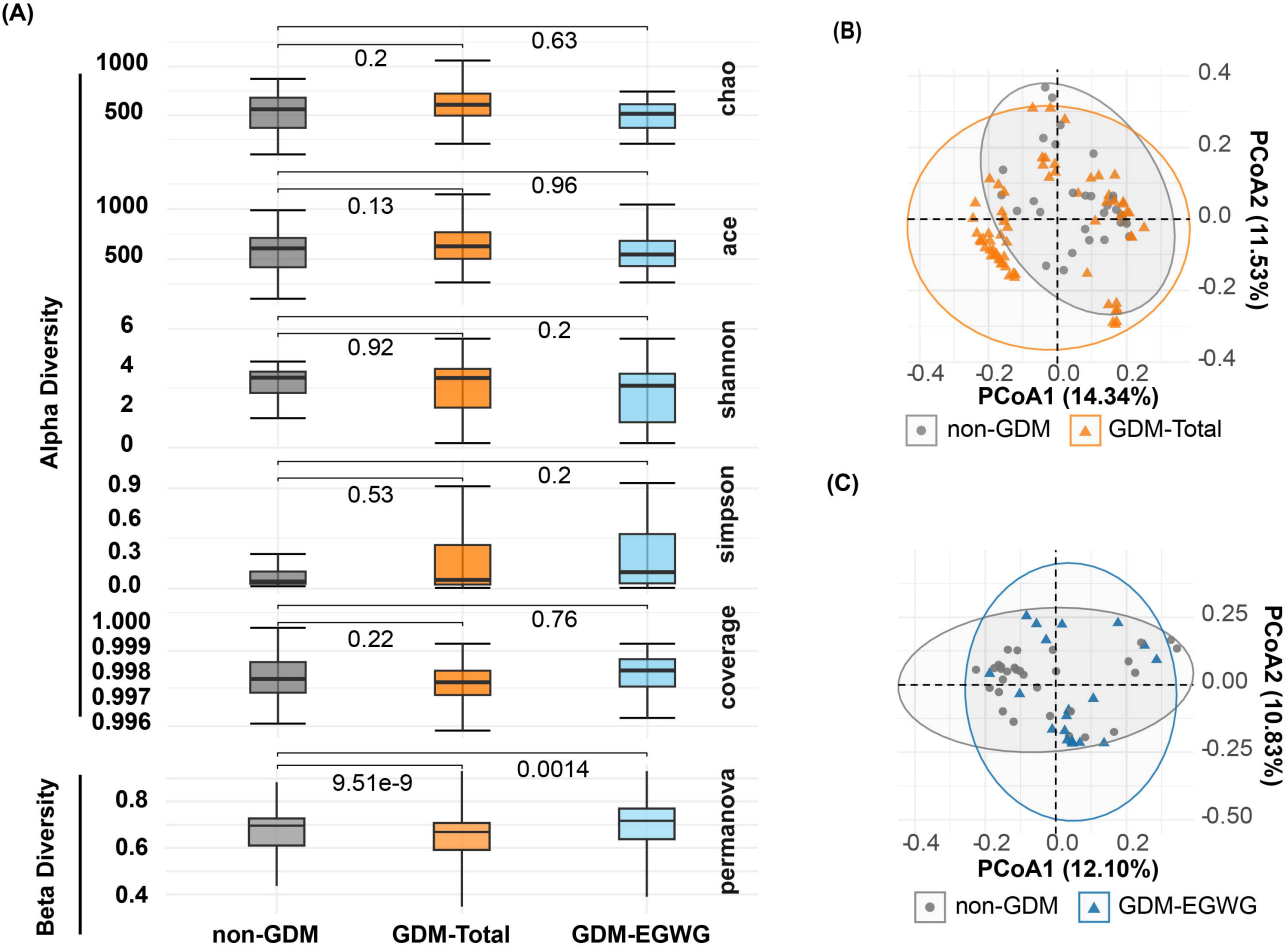
Alpha and beta diversity of gut microbiota in offspring. **(A)** Comparisons of Chao, ACE, Shannon, Simpson, and Coverage diversity indices among offspring born to mothers with non-GDM, GDM-Total, and GDM+EGWGgroups, with PERMANOVA results indicating beta diversity differences between groups; **(B, C)** Principal Coordination Analysis (PCoA) based on unweighted UniFrac distances, illustrating microbial community structures in offspring born to mothers in the non-GDM group compared to those in the GDM-Total **(B)** and GDM+EGWG **(C)** groups.

No significant differences in alpha diversity metrics were observed among the non-GDM, GDM-Total, and GDM-EGWG groups. Specifically, richness indices such as Chao1 (*P* = 0.2 for non-GDM vs. GDM-Total; *P* = 0.63 for non-GDM vs. GDM-EGWG) and ACE (*P* = 0.13 for non-GDM vs. GDM-Total; *P* = 0.96 for non-GDM vs. GDM-EGWG) showed no significant differences (**Figure 2A**). Similarly, evenness indices, including Shannon (*P* = 0.92 for non-GDM vs. GDM-Total; *P* = 0.2 for non-GDM vs. GDM-EGWG) and Simpson (*P* = 0.53 for non-GDM vs. GDM-Total; *P* = 0.2 for non-GDM vs. GDM-EGWG), did not differ significantly among these groups. Coverage also did not show significant differences (*P* = 0.22 for non-GDM vs. GDM-Total; *P* = 0.76 for non-GDM vs. GDM-EGWG).

In contrast, beta diversity analysis using PERMANOVA indicated significant differences in microbial community structure. The non-GDM and GDM-Total groups displayed substantial differences (*P* = 9.51e-9), as did the non-GDM and GDM-EGWG groups (*P* = 0.0014), indicating distinct microbial community compositions associated with GDM status and excess gestational weight gain (EGWG) (**Figure 2A**).

**Figure 3:**
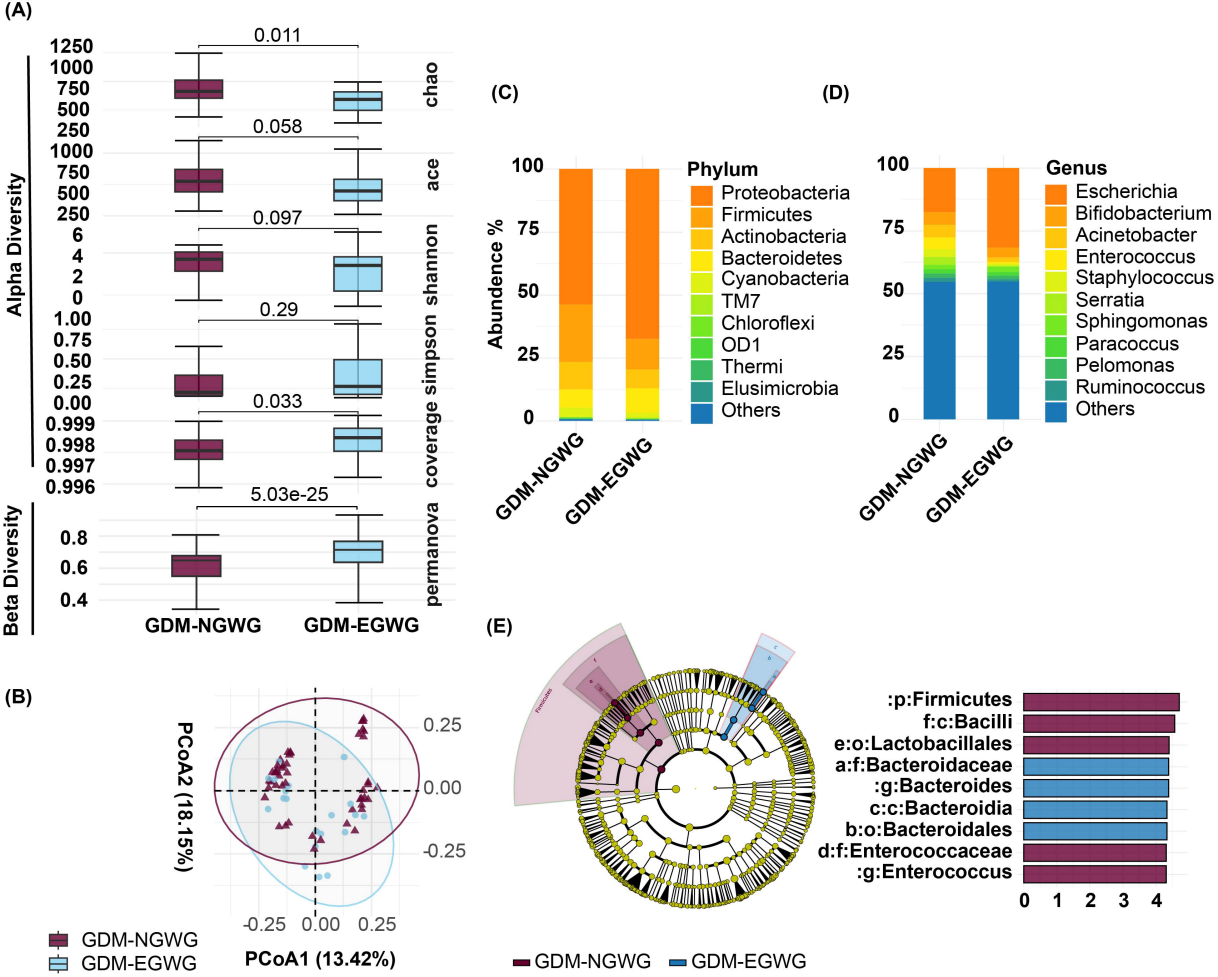
Gut Microbiota Analysis in Offspring from GDM+NGWG and GDM+EGWG Groups. **(A)** Alpha diversity indices (Chao1, ACE, Shannon, Simpson, and Coverage) and beta diversity (PERMANOVA) comparing microbiota diversity between GDM-NGWG and GDM-EGWG groups, with P-values indicating significance.; **(B)** Principal Coordinate Analysis (PCoA) based on unweighted UniFrac distances, showing microbial community differences between the groups; **(C, D)** Relative abundances of microbial phyla **(C)** and genera **(D)** in offspring from the GDM+NGWG and GDM+EGWG groups; **(E)** LEfSe analysis, with a cladogram (left) showing differentially abundant taxa and an LDA score plot (right) displaying the effect size of these taxa, with purple indicating enrichment in the GDM+NGWG group and blue in the GDM+EGWG group.

Within the GDM subgroups, comparisons between GDM+EGWG and GDM+NGWG revealed no significant differences in alpha diversity metrics. Richness indices (Chao1: *P* = 0.011; ACE: *P* = 0.058) and evenness indices (Shannon: *P* = 0.097; Simpson: *P* = 0.29) showed only minor differences, and coverage was similar between these groups (*P* = 0.033) (**Figure 3A**). In contrast, beta diversity analysis identified significant variations in microbial community structure between GDM+EGWG and GDM+NGWG (*P* = 5.03e-25), suggesting that excess gestational weight gain (EGWG) may influence microbial composition independently of overall GDM status (**Figure 3A**).

### Differential bacteria and functional predictions

**Figure 4:**
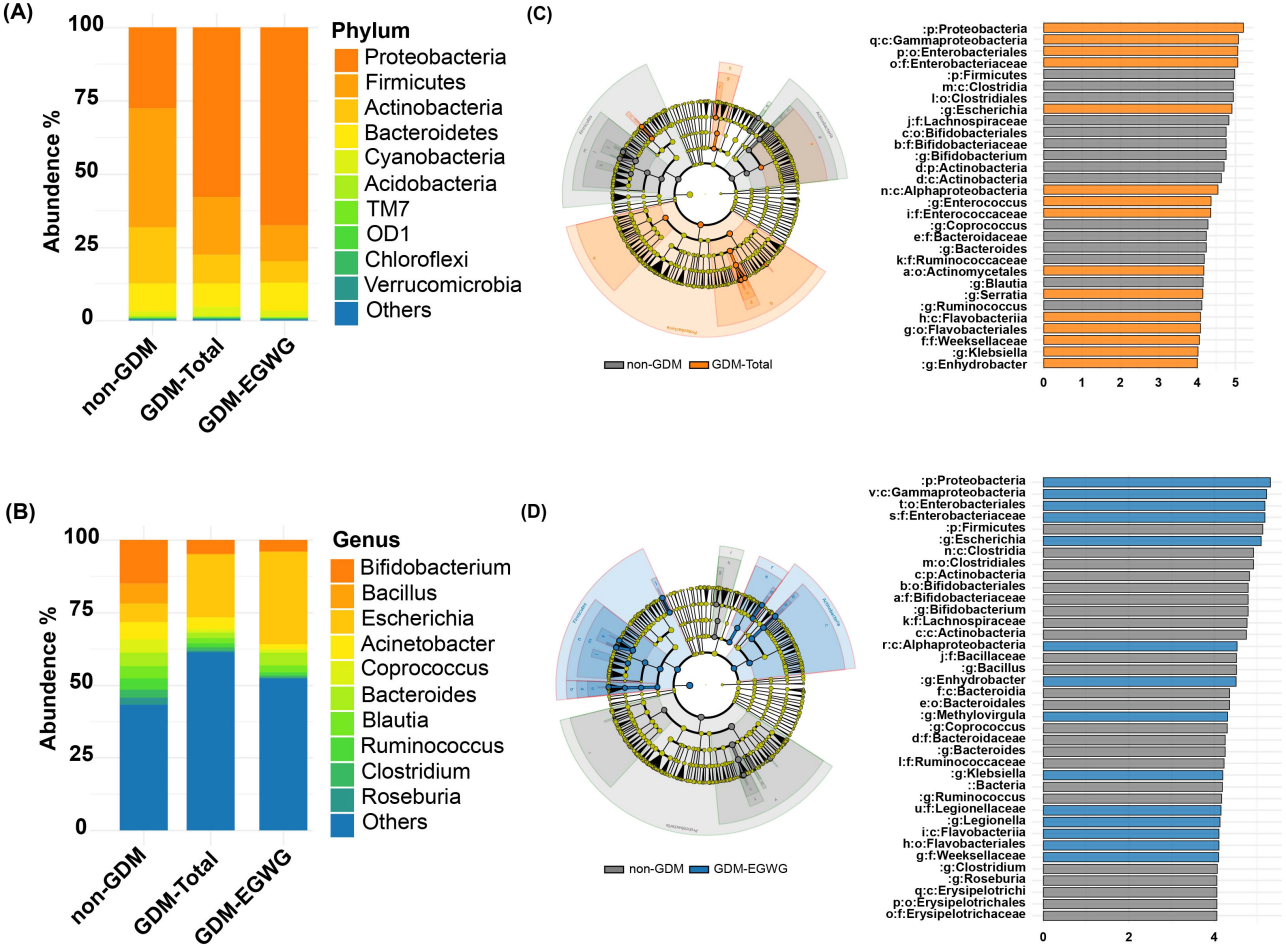
Differential Bacteria in GDM-Total and GDM+EGWG groups compared to non-GDM. **(A-B)** Relative proportions of abundant microbes at the phylum **(A)** and genus **(B)** levels in offspring born to mothers in the non-GDM, GDM-Total, and GDM+EGWG groups; **(C, D)** LEfSe analysis of gut microbiota in offspring born to mothers in the GDM-Total versus non-GDM groups **(C)** and the GDM+EGWG versus non-GDM groups **(D)**. The left side of each panel displays a cladogram (branch diagram) that illustrates the taxonomic hierarchy of bacterial taxa with significant differences in abundance between groups, colored according to the group in which they are enriched. The right side shows a corresponding histogram (LDA score plot) where the length of each bar represents the LDA score, indicating the effect size of each differentially abundant taxon, with colors denoting enrichment in the respective group (orange for GDM-Total, blue for GDM+EGWG, and gray for non-GDM).

Differential bacteria analysis showed that the main phyla included *Actinobacteria, Bacteroidetes, Firmicutes, and Proteobacteria*, with significant enrichments and depletions observed across the groups. Specifically, *Proteobacteria* were enriched while *Firmicutes* were depleted in GDM-Total and GDM+EGWG groups compared to non-GDM (**Figures 4A, C, D**). Notable genus-level changes included enrichment of *Escherichia* and *Klebsiella*, and depletion of *Bacteroides, Bifidobacterium Coprococcus,Ruminococcus* among GDM-Total and GDM+EGWG groups (**Figures 4B, C, D**). Further, LEfSe analysis identified 30 differential bacteria taxa between GDM-Total and healthy control groups, which increased to 38 between GDM+EGWG and non-GDM groups, highlighting more pronounced microbial shifts associated with EGWG.

The newborn gut microbiota composition in the GDM+EGWG groups was also significantly different from that of GDM+NGWG groups, with *Enterococcus* being significantly enriched. (**Figure 3**, appendix for LDA values in **Supplementary Table S1**). Maternal gestational weight gain significantly altered the β-diversity of neonatal microbiota, demonstrating distinct microbial structures based on the degree of weight gain (*P* < 0.001, **Figure 3**). PICRUSt2 predictions indicated that pathways related to energy production and lipid metabolism were particularly affected, suggesting metabolic adaptations linked to altered gut microbiota in GDM affected pregnancies.

### Network analysis of gut microbiota in GDM-total and GDM+EGWG conditions

**Figure 5:**
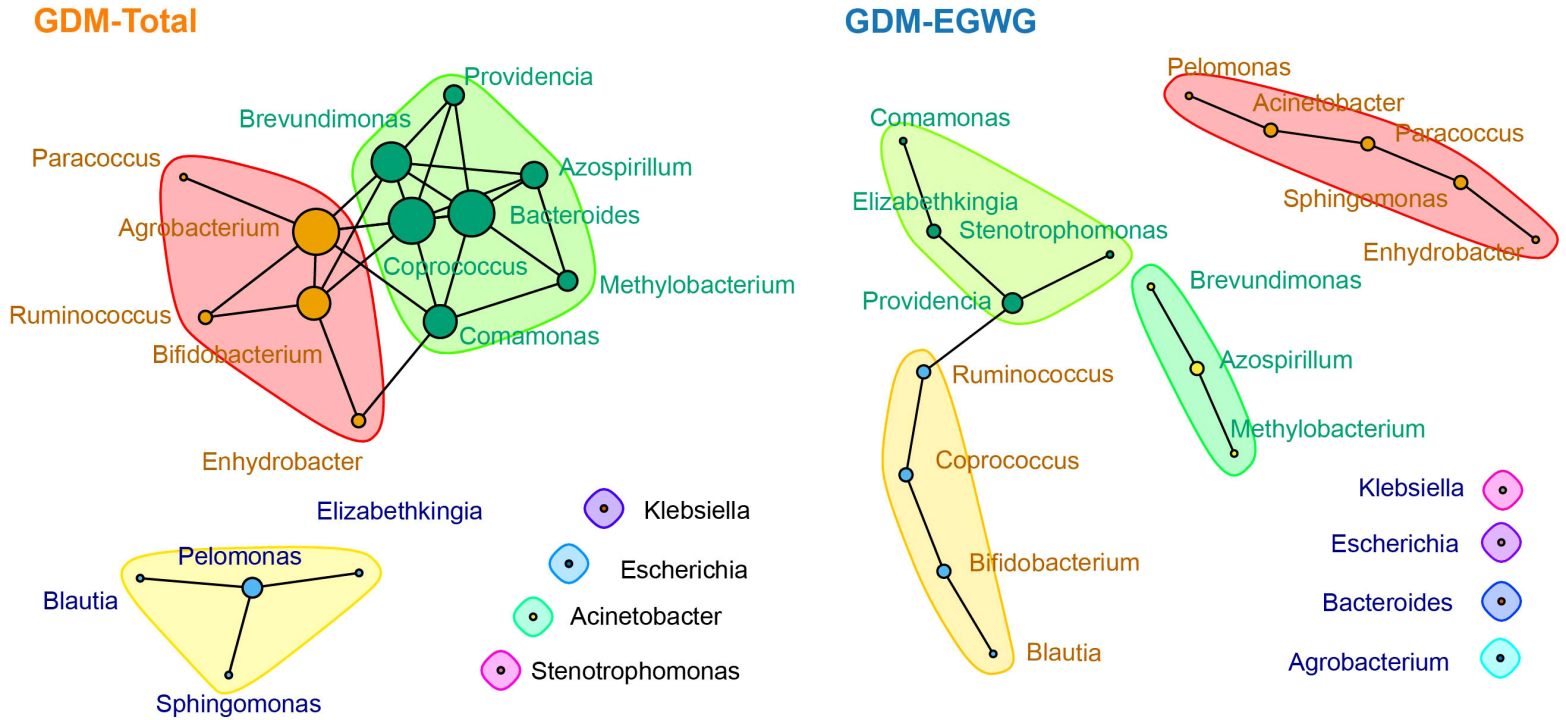
Ecological association networks in the GDM-Total and GDM-EGWG groups. Nodes represent the top 20 genera by abundance, with edges illustrating the predicted ecological interactions between these genera. The size of each node corresponds to the strength of interaction, with larger nodes indicating more central or influential genera within the network. Different color blocks highlight distinct modularity classes, indicating clusters of genera that are more closely associated with each other within each group’s microbial community network.

In the ecological association network analysis of the gut microbiota in GDM-Total and GDM+EGWG groups (**Figure 5**), using the top 20 most abundant genera, we observed a notable difference in the number of edges representing interactions between genera. The GDM-Total group’s network depicted 29 edges, suggesting a denser web of microbial interactions, whereas the GDM+EGWG group exhibited a sparser network with 13 edges. In the GDM-Total group, *Providencia, Acinetobacter*, and *Azospirillum* emerged as the top three genera based on network centrality measures. In contrast, the GDM+EGWG group’s top three genera were *Agrobacterium, Bacteroides*, and *Coprococcus*. The network’s modularity suggests subgroupings within the microbial community, which may have functional implications in the context of GDM and EGWG.

### Linear correlation analysis between gestational weight gain and key gut bacterial taxa in neonates

**Figure 6:**
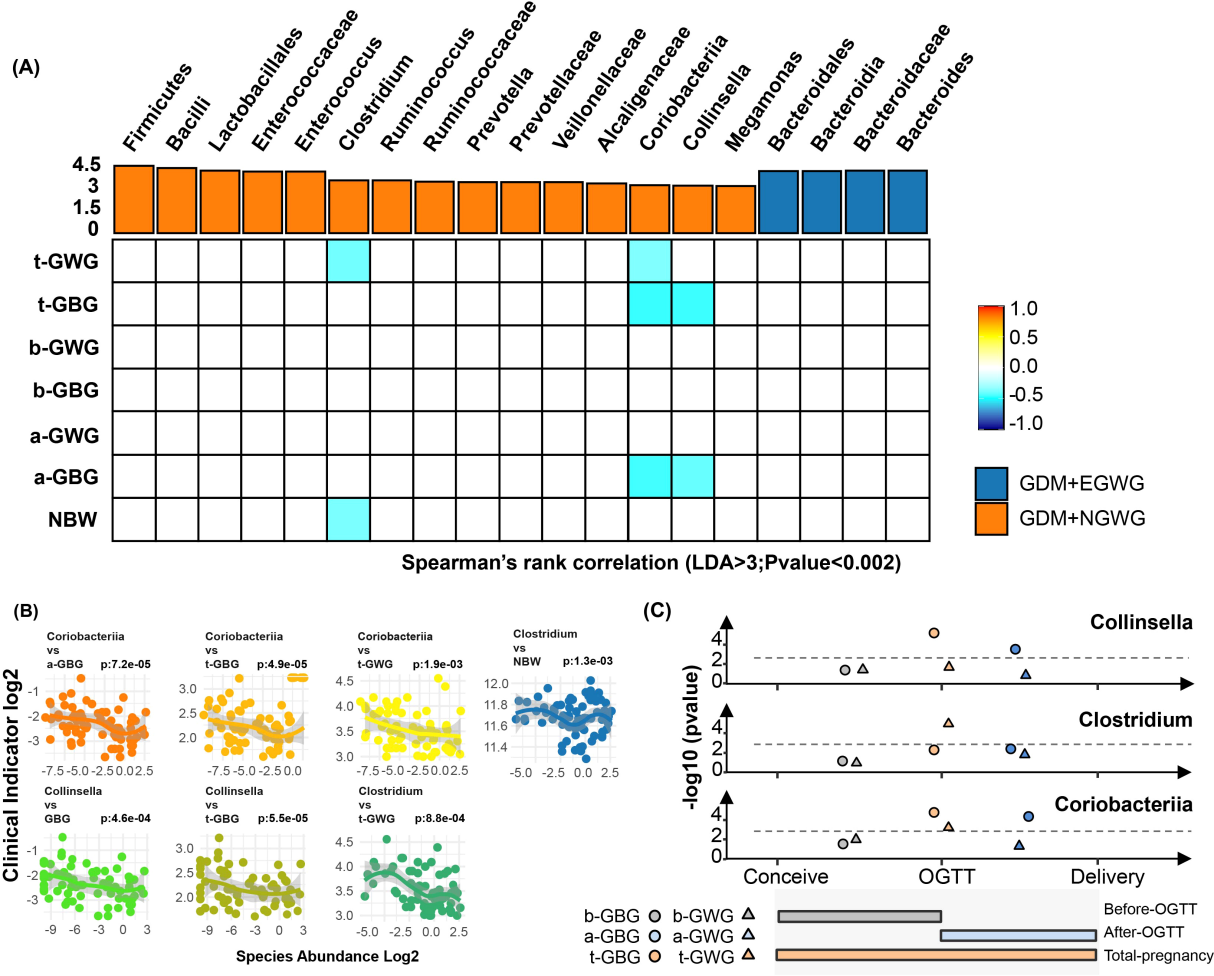
Associations of Pregnancy weight gain with altered gut bacteria. **(A)** Heatmap showing the Spearman’s rank correlation coefficients between six pregnancy weight gain indices (t-GWG, t-GBG, b-GWG, b-GBG, a-GWG, a-GBG, NBW) and 21 gut bacterial taxa. Positive correlations are indicated in orange, and negative correlations in blue, with color intensity reflecting the strength of the correlation (*P* < 0.05). **(B)** Scatter plots illustrating significant correlations between key bacterial taxa (*Coriobacteriia, Collinsella, Clostridium*) and various pregnancy weight gain indices, highlighting relationships with specific time points (before OGTT, after OGTT, total pregnancy); **(C)** Line graphs summarizing the correlations of Collinsella, Clostridium, and Coriobacteriia with clinical indicators at different pregnancy stages. Colors represent the timing of weight gain: brown for pre-pregnancy to OGTT, blue for OGTT to pre-delivery, and orange for pre-pregnancy to pre-delivery. Shapes indicate the specific weight gain indices: circles for GBG and triangles for GWG. The gray line represents the 0.002 significance threshold.

To investigate the influence of gestational weight gain on specific gut microbiota, Spearman correlation analysis was conducted (**Figure 6**). Significant correlations were identified: *Clostridium* was negatively correlated with total gestational weight gain (t-GWG) and newborn birth weight (NBW); The *Coriobacteriaceae* family along with related orders and classes, showed negative correlated with t-GWG, total gestational BMI gain (t-GBG), and BMI gain after OGTT (a-GBG); Additionally, *Collinella* exhibited negative correlations with t-GBG and a-GBG (*P*<0.002) (attachment for detailed data **Supplementary Table S2**).

## Discussion

The neonatal period is a critical window for immune maturation, during which the gut microbiota plays a pivotal role in shaping health outcomes in both childhood and adulthood. The maternal influence on the neonatal gut microbiota is paramount, often outweighing the impact of the delivery environment. Factors such as gestational age at birth, mode of delivery, and initial feeding method are significant contributors (Yin et al., 2023).

Interestingly, the maternal’s influence on the newborn’s gut microbiota may begin even earlier, during the prenatal period (Perez-Munoz et al., 2017; Lu et al., 2024; Wang et al., 2020). While parental genetics do contribute to the composition of the newborn’s gut microbiota, the intrauterine environment and the mother’s health status during pregnancy have a more profound impact. The precise mechanisms underlying this influence remain unclear, but proposed hypotheses include vertical transmission of maternal bacteria to the newborn’s gut and the presence if maternal immune-active substances in the placenta or umbilical blood that may modify specific ecological niches (Caprara et al., 2024).

In this study, a cohort of 98 mother-infant pairs was included, with all infants delivered vaginally, and stool samples collected within 24 hours of birth. The results indicated a lower relative abundance of *Firmicutes* and an elevated presence of *Proteobacteria* at the phylum level in the GDM-Total group compared to the healthy control group. At the genus level, a reduction in beneficial taxa such as *Bifidobacterium*, *Coprococcus*, and *Ruminococcus* was observed, alongside an increase in potentially opportunistic taxa, including *Escherichia* and *Klebsiella*. In a related study (Su et al., 2018), examined the gut microbiota of infants delivered by cesarean section and reported a significant increase in *Proteobacteria* and a decrease in *Bifidobacterium* in infants born to mothers with GDM compared to controls. Similarly Crusell et al. (2018), observed an increase in *Proteobacteria* and a reduction in *Collinsella* and *Actinobacteria* in 9-month-old infants exposed to GDM *in utero*. These findings collectively suggest that maternal GDM status may impact the stability of the infant gut microbiome. However, discrepancies in findings exist across studies, likely due to variations in sampling time, detection methods, and study populations. For instance (Chen et al., 2021)and (Zhu et al., 2022)reported an increased abundance of *Firmicutes* and a decreased abundance of *Proteobacteria* in the infant gut microbiome in GDM-exposed newborns.

Metabolic irregularities in GDM mothers often extend beyond glucose metabolism disorders, with excessive gestational weight gain (EGWG) being a common issue. A meta-analysis by Viecceli et al. reported that the incidence of EGWG in GDM mothers is 39.5% (Viecceli et al., 2017). Strict weight control is known to significantly improve outcomes for GDM mothers and their offspring, reducing the risk of complications such as gestational hypertension, cesarean sections, and macrosomia. Current obstetric practices increasingly emphasize weight management in GDM mothers, particularly to prevent EGWG (Santos Monteiro et al., 2023; Yew et al., 2021; Wu et al., 2023; Schenk et al., 2024). However, the combined impact of GDM and EGWG on neonatal gut microbiota remains underexplored.

Our analysis indicated that the GDM+EGWG group exhibited a greater number of differentially abundant genera compared to the non-GDM group, suggesting that EGWG intensifies the disruption of gut microbiota in offspring born to GDM mothers. The ecological network differences observed between the GDM-Total and GDM+EGWG groups emphasize the significant impact of maternal conditions on the structure of the gut microbiota. The denser network in the GDM-Total group suggests a disrupted yet highly interactive microbial ecosystem, potentially reflecting adaptations to metabolic stress. In contrast, the sparser network in the GDM+EGWG group indicates reduced microbial interconnectivity, which may undermine microbiota resilience and overall health stability. This study found that neonates born to GDM mothers with EGWG, compared to those with NGWG, exhibited significant differences in the composition of their gut microbiota.

To understand the potential “shaping” and “driving” effects of maternal gestational weight gain on neonatal gut microbiota, we conducted linear correlation analyses between gestational weight gain indicators and key differential bacterial taxa. Taxa that showed significant differences between the GDM+EGWG and GDM+NGWG groups (LDA > 3.0, relative abundance > 0.5%) were identified as “key taxa.” Notably, the relative abundance of *Clostridium/Coriobacteriaceae/Collinsella* in the neonatal gut was inversely correlated with t-GWG or t-GBG, indicating that maternal weight gain in GDM pregnancies may shape the characteristics on the neonatal gut microbiota.

Pregnant women are typically diagnosed with GDM between 24 and 28 weeks of gestation, after which strict weight management usually commences. This raises the question: Can weight management still positively influence perinatal outcomes after this point? Barnes et al. reported that avoiding excessive weight gain throughout pregnancy and after OGTT diagnosis benefits glycemic control in pregnant women and reduces the incidence of macrosomia in newborns (Barnes et al., 2020). Zheng et al. found that excessive total gestational weight gain in mothers not only raises the risk of macrosomia in newborns but also increases the likelihood of childhood obesity up to 40 months postpartum (Zheng et al., 2024).However, EGWG before GDM diagnosis does not independently elevate the risk of childhood obesity. These observations underscore the importance of weight management during late pregnancy in shaping neonatal outcomes. Our research shows that gestational weight gain after GDM diagnosis is negatively correlated with the abundance of *Coriobacteriaceae/Collinsella* in newborns, thereby affecting their health status.

The above “key bacterial taxa” identified, such as *Clostridium, Coriobacteriaceae*, and *Collinsella*, have direct implications on metabolism and immune function, both of which are closely associated with newborn macrosomia or childhood obesity. Petersen et al. described *Clostridium’s* ability to downregulate the expression of the cell surface molecule CD36, which facilitates the uptake of fatty acids in humans. By hindering lipid absorption in the intestines, *Clostridium* may act as an “Anti-Obesity Microorganism” (Petersen et al., 2019).Our findings corroborate this, showing that lighter gestational weight gain correlates with higher *Clostridium* abundance in the newborn’s gut. We also found an inverse relationship between *Clostridium* abundance and birth weight, further suggesting its potential anti-obesity effects. *Coriobacteriaceae*, important producers of short-chain fatty acids (SCFAs), contribute to intestinal barrier integrity, regulate immune responses, and maintain energy homeostasis. They also inhibit fat storage in adipose tissue and activate the AMPK pathway in the liver, displaying anti-obesity bioactivity (Yi et al., 2021; Reigstad et al., 2015; Cani et al., 2008; Riviere et al., 2016). *Collinsella*, a part of the *Coriobacteriaceae* family, synthesizes ursodeoxychlic acid (UDCA), which promotes thermogenesis in brown adipose tissue, thus potentially suppressing obesity (Liu et al., 2011; Wei et al., 2020). Early colonization by *Coriobacteriaceae* and *Collinsella* is advantageous for establishing a healthy gut ecosystem in newborns (He et al., 2022).

Stringent weight control is essential after diagnosing GDM to improve the neonatal gut microbiota effectively and should not be disregarded as “too late.” These findings provide new insights for precise prevention and management of GDM.The deepening degree of gestational weight gain in GDM pregnancies corresponds with a decline in the relative abundance of various “anti-obesity” bacteria and microbial groups that benefit gut microbial homeostasis in neonatal gut microbiota, thereby increasing health risks. Thus, maternal weight gain during pregnancy plays a significant role in influencing infant and child growth and development by modulating the characteristics of neonatal gut microbiota. Although weight management should ideally begin before and early in pregnancy, stringent weight control after GDM diagnosis has been shown to have a positive impact on perinatal outcomes (Yew et al., 2021). This study suggests that, in the clinical management of GDM, attention to both blood glucose and weight control may be beneficial to perinatal outcomes, potentially reflected in improvements in neonatal gut microbiota.

This study uniquely addresses the impact of maternal metabolic phenotype aggregation (GDM with EGWG) on neonatal gut microbiota. Given the clinical heterogeneity of GDM, stratified analyses based on the presence of other metabolic abnormalities, such as EGWG, are essential for precise clinical interventions. Furthermore, this study explores the influence of gestational weight gain on neonatal gut microbiota by examining distinct periods: the entire gestation, and the periods before and after a GDM diagnosis. The application of high-throughput sequencing to analyze neonatal gut microbiota emerges as a valuable tool to assess the efficacy of clinical interventions in GDM.

The study acknowledges certain limitations. First, the absence of metabolomics detection methods precludes the direct assessment of metabolite levels associated with the neonatal gut microbiota, limiting our ability to fully understand the influence of gestational weight gain on offspring. Additionally, the study’s participants were drawn from a single medical institution, which may affect the generalizability of the findings. Further multicenter research is needed to validate and expand upon these results. Finally, all participants had normal pre-pregnancy BMI, which restricts the generalizability of our findings. Future research should include women with varying BMI categories to more comprehensively assess the effects of maternal BMI on the neonatal gut microbiome.

## Conclusion

This study has identified a disruption in the gut microbiota homeostasis of newborns born to mothers with GDM,with the condition of EGWG in GDM mothers further exacerbating these microbial perturbations. Inverse correlations were found between t-GWG/GBG and a-GBG and the relative abundance of key differentially abundant bacterial taxa (*Clostridium, Coriobacteriaceae, Collinsella*),which are beneficial for maintaining normal metabolic and immune functions in GDM offspring. Once GDM is diagnosed in pregnant women, weight management should be given high priority in clinical practice. The 16S rRNA sequencing analysis of neonatal gut microbiota offers insights into the effectiveness of weight management and other clinical interventions, presenting substantial implications for enhancing clinical management and perinatal health outcomes.

## Data Availability

The data presented in the study are deposited in the NCBI repository, accession number PRJNA1108428.
