# Supplementary material for: Gestational diabetes-combined excess weight gain exacerbates gut microbiota dysbiosis in newborns, associated with reduced abundance of Clostridium, Coriobacteriaceae, and Collinsella
Source: Front Cell Infect Microbiol. 2024 Dec 13;14:1496447. doi: 10.3389/fcimb.2024.1496447 (PMC11670820; doi:10.3389/fcimb.2024.1496447)
Supplement: Supplementary file 1 [file Table1.docx]

**Gestational diabetes-combineded excess weight gain exacerbates gut microbiota dysbiosis in newborns, associated with reduced abundance of *Clostridium*, *Coriobacteriaceae*, and *Collinsella***

Supplementary Material

# Supplementary Data

***Table S1:*LDA values between non GDM, GDM Total, and GDM+EGWG groups**

| species | Group | LDA |
| --- | --- | --- |
| *Bacteroides* | non-GDM | 4.24 |
| *Ruminococcaceae* | non-GDM | 4.18 |
| *Bifidobacteriaceae* | non-GDM | 4.76 |
| *Bifidobacterium* | non-GDM | 4.75 |
| *Bacteroidaceae* | non-GDM | 4.24 |
| *Firmicutes* | non-GDM | 4.98 |
| *Blautia* | non-GDM | 4.16 |
| *Lachnospiraceae* | non-GDM | 4.83 |
| *Bifidobacteriales* | non-GDM | 4.76 |
| *Ruminococcus* | non-GDM | 4.12 |
| *Actinobacteria* | non-GDM | 4.63 |
| *Clostridiales* | non-GDM | 4.95 |
| *Clostridia* | non-GDM | 4.95 |
| *Coprococcus* | non-GDM | 4.28 |
| *Actinobacteria* | non-GDM | 4.70 |
| *Weeksellaceae* | GDM-Total | 4.05 |
| *Actinomycetales* | GDM-Total | 4.17 |
| *Enterococcaceae* | GDM-Total | 4.36 |
| *Proteobacteria* | GDM-Total | 5.21 |
| *Alphaproteobacteria* | GDM-Total | 4.55 |
| *Flavobacteriia* | GDM-Total | 4.08 |
| *Enterobacteriaceae* | GDM-Total | 5.06 |
| *Enterococcus* | GDM-Total | 4.36 |
| *Gammaproteobacteria* | GDM-Total | 5.08 |
| *Serratia* | GDM-Total | 4.15 |
| *Enterobacteriales* | GDM-Total | 5.06 |
| *Klebsiella* | GDM-Total | 4.02 |
| *Flavobacteriales* | GDM-Total | 4.08 |
| *Escherichia* | GDM-Total | 4.91 |
| *Enhydrobacter* | GDM-Total | 4.01 |
| *Weeksellaceae* | GDM-EGWG | 4.10 |
| *Enterobacteriales* | GDM-EGWG | 5.18 |
| *Proteobacteria* | GDM-EGWG | 5.31 |
| *Methylovirgula* | GDM-EGWG | 4.31 |
| *Alphaproteobacteria* | GDM-EGWG | 4.54 |
| *Flavobacteriia* | GDM-EGWG | 4.11 |
| *Enterobacteriaceae* | GDM-EGWG | 5.18 |
| *Gammaproteobacteria* | GDM-EGWG | 5.22 |
| *Legionellaceae* | GDM-EGWG | 4.15 |
| *Klebsiella* | GDM-EGWG | 4.20 |
| *Flavobacteriales* | GDM-EGWG | 4.11 |
| *Escherichia* | GDM-EGWG | 5.10 |
| *Legionella* | GDM-EGWG | 4.13 |
| *Enhydrobacter* | GDM-EGWG | 4.51 |

***Table S2:*the influence of gestational weight gain on specific gut microbiota**

| GDM | Meta | pvalue | rho |
| --- | --- | --- | --- |
| t-GBG | Coriobacteriia | 4.9002060377543e-05 | -0.471682989 |
| t-GBG | Coriobacteriaceae | 4.9002060377543e-05 | -0.471682989 |
| t-GBG | Coriobacteriales | 4.9002060377543e-05 | -0.471682989 |
| t-GBG | Collinsella | 5.52367419251528e-05 | -0.468813994 |
| a-GBG | Coriobacteriia | 7.24471856305271e-05 | -0.462220718 |
| a-GBG | Coriobacteriaceae | 7.24471856305271e-05 | -0.462220718 |
| a-GBG | Coriobacteriales | 7.24471856305271e-05 | -0.462220718 |
| a-GBG | Collinsella | 0.000460019 | -0.41330352 |
| t-GWG | Clostridium | 0.000885181 | -0.39403787 |
| NBW | Clostridium | 0.001340329 | -0.381199599 |
| t-GWG | Coriobacteriia | 0.001959126 | -0.368984102 |
| t-GWG | Coriobacteriaceae | 0.001959126 | -0.368984102 |
| t-GWG | Coriobacteriales | 0.001959126 | -0.368984102 |
| t-GWG | Bacilli | 0.097162254 | -0.202818949 |
| t-GWG | Bacteroidia | 0.133798173 | -0.183670303 |
| t-GWG | Alcaligenaceae | 0.01797728 | -0.286205941 |
| t-GWG | Bacteroidaceae | 0.131996607 | -0.184511161 |
| t-GWG | Enterococcaceae | 0.063437023 | -0.226352613 |
| t-GWG | Prevotellaceae | 0.162664975 | -0.171231049 |
| t-GWG | Ruminococcaceae | 0.05008305 | -0.238574556 |
| t-GWG | Veillonellaceae | 0.069699281 | -0.221329509 |
| t-GWG | Bacteroides | 0.131996607 | -0.184511161 |
| t-GWG | Collinsella | 0.006293391 | -0.328169414 |
| t-GWG | Enterococcus | 0.075691269 | -0.216851558 |
| t-GWG | Megamonas | 0.317216918 | -0.123111557 |
| t-GWG | Prevotella | 0.090187837 | -0.20708255 |
| t-GWG | Ruminococcus | 0.002466316 | -0.361339932 |
| t-GWG | Bacteroidales | 0.133798173 | -0.183670303 |
| t-GWG | Lactobacillales | 0.016349602 | -0.290249147 |
| t-GWG | Firmicutes | 0.010133908 | -0.309837334 |
| t-GBG | Bacilli | 0.01684915 | -0.288972877 |
| t-GBG | Bacteroidia | 0.155904449 | -0.173986906 |
| t-GBG | Alcaligenaceae | 0.063191296 | -0.226557728 |
| t-GBG | Bacteroidaceae | 0.036212385 | -0.25451908 |
| t-GBG | Enterococcaceae | 0.010856261 | -0.307094516 |
| t-GBG | Prevotellaceae | 0.11474982 | -0.193038549 |
| t-GBG | Ruminococcaceae | 0.002777038 | -0.357326921 |
| t-GBG | Veillonellaceae | 0.415890155 | -0.100270098 |
| t-GBG | Bacteroides | 0.036212385 | -0.25451908 |
| t-GBG | Clostridium | 0.012239264 | -0.302258109 |
| t-GBG | Enterococcus | 0.011192235 | -0.305872478 |
| t-GBG | Megamonas | 0.453812551 | -0.092356445 |
| t-GBG | Prevotella | 0.063351727 | -0.226423739 |
| t-GBG | Ruminococcus | 0.046094284 | -0.242741798 |
| t-GBG | Bacteroidales | 0.155904449 | -0.173986906 |
| t-GBG | Lactobacillales | 0.011424836 | -0.305044954 |
| t-GBG | Firmicutes | 0.014679029 | -0.294775622 |
| b-GBG | Bacilli | 0.315365104 | 0.123579932 |
| b-GBG | Bacteroidia | 0.899279707 | 0.015638018 |
| b-GBG | Coriobacteriia | 0.072302129 | 0.219347945 |
| b-GBG | Alcaligenaceae | 0.44062591 | 0.095064468 |
| b-GBG | Bacteroidaceae | 0.444695369 | 0.094224007 |
| b-GBG | Coriobacteriaceae | 0.072302129 | 0.219347945 |
| b-GBG | Enterococcaceae | 0.374090353 | -0.109492806 |
| b-GBG | Prevotellaceae | 0.515761823 | -0.080170351 |
| b-GBG | Ruminococcaceae | 0.479829187 | 0.087137411 |
| b-GBG | Veillonellaceae | 0.389065001 | 0.10612179 |
| b-GBG | Bacteroides | 0.444695369 | 0.094224007 |
| b-GBG | Clostridium | 0.964731054 | -0.005463409 |
| b-GBG | Collinsella | 0.250704683 | 0.141211714 |
| b-GBG | Enterococcus | 0.319399187 | -0.122561845 |
| b-GBG | Megamonas | 0.418248831 | 0.099766219 |
| b-GBG | Prevotella | 0.530319192 | -0.077418825 |
| b-GBG | Ruminococcus | 0.392369586 | 0.105388364 |
| b-GBG | Bacteroidales | 0.899279707 | 0.015638018 |
| b-GBG | Coriobacteriales | 0.072302129 | 0.219347945 |
| b-GBG | Lactobacillales | 0.639299584 | 0.057860666 |
| b-GBG | Firmicutes | 0.129779564 | 0.185557949 |
| a-GBG | Bacilli | 0.009515225 | -0.312325239 |
| a-GBG | Bacteroidia | 0.270343931 | -0.135565127 |
| a-GBG | Alcaligenaceae | 0.03131623 | -0.2613819 |
| a-GBG | Bacteroidaceae | 0.034310885 | -0.25708651 |
| a-GBG | Enterococcaceae | 0.209639573 | -0.154083648 |
| a-GBG | Prevotellaceae | 0.367450715 | -0.111013517 |
| a-GBG | Ruminococcaceae | 0.010021138 | -0.310280775 |
| a-GBG | Veillonellaceae | 0.211453342 | -0.153479532 |
| a-GBG | Bacteroides | 0.034310885 | -0.25708651 |
| a-GBG | Clostridium | 0.021593571 | -0.278238109 |
| a-GBG | Enterococcus | 0.240079101 | -0.144393093 |
| a-GBG | Megamonas | 0.175182182 | -0.166343491 |
| a-GBG | Prevotella | 0.219529234 | -0.150833168 |
| a-GBG | Ruminococcus | 0.100646984 | -0.200777781 |
| a-GBG | Bacteroidales | 0.270343931 | -0.135565127 |
| a-GBG | Lactobacillales | 0.036293931 | -0.254411511 |
| a-GBG | Firmicutes | 0.008278849 | -0.317751665 |
| NBW | Bacilli | 0.93745015 | -0.009696044 |
| NBW | Bacteroidia | 0.02046517 | -0.280593597 |
| NBW | Coriobacteriia | 0.30028811 | -0.127460992 |
| NBW | Alcaligenaceae | 0.237600292 | -0.145149129 |
| NBW | Bacteroidaceae | 0.088221406 | -0.208331345 |
| NBW | Coriobacteriaceae | 0.30028811 | -0.127460992 |
| NBW | Enterococcaceae | 0.01237073 | -0.301823403 |
| NBW | Prevotellaceae | 0.467867165 | -0.089517484 |
| NBW | Ruminococcaceae | 0.033740049 | -0.257880422 |
| NBW | Veillonellaceae | 0.391035214 | 0.105684074 |
| NBW | Bacteroides | 0.088221406 | -0.208331345 |
| NBW | Collinsella | 0.156842852 | -0.173599158 |
| NBW | Enterococcus | 0.013163381 | -0.299284024 |
| NBW | Megamonas | 0.136742113 | 0.182314484 |
| NBW | Prevotella | 0.555323721 | -0.072778287 |
| NBW | Ruminococcus | 0.002546607 | -0.360261488 |
| NBW | Bacteroidales | 0.02046517 | -0.280593597 |
| NBW | Coriobacteriales | 0.30028811 | -0.127460992 |
| NBW | Lactobacillales | 0.383511714 | -0.107362695 |
| NBW | Firmicutes | 0.471595192 | -0.088772248 |
| b-GWG | Bacilli | 0.484977728 | -0.08612278 |
| b-GWG | Bacteroidia | 0.245256106 | -0.14283126 |
| b-GWG | Coriobacteriia | 0.012371373 | -0.301821285 |
| b-GWG | Alcaligenaceae | 0.27272887 | -0.134898453 |
| b-GWG | Bacteroidaceae | 0.157393397 | -0.173372472 |
| b-GWG | Coriobacteriaceae | 0.012371373 | -0.301821285 |
| b-GWG | Enterococcaceae | 0.545483821 | -0.074592081 |
| b-GWG | Prevotellaceae | 0.120113282 | -0.190287511 |
| b-GWG | Ruminococcaceae | 0.068297014 | -0.222421773 |
| b-GWG | Veillonellaceae | 0.232139342 | -0.146834152 |
| b-GWG | Bacteroides | 0.157393397 | -0.173372472 |
| b-GWG | Clostridium | 0.727858845 | -0.042975439 |
| b-GWG | Collinsella | 0.011882365 | -0.303458955 |
| b-GWG | Enterococcus | 0.550241332 | -0.073713178 |
| b-GWG | Megamonas | 0.242827069 | -0.143561218 |
| b-GWG | Prevotella | 0.091386456 | -0.206331828 |
| b-GWG | Ruminococcus | 0.111622933 | -0.19468829 |
| b-GWG | Bacteroidales | 0.245256106 | -0.14283126 |
| b-GWG | Coriobacteriales | 0.012371373 | -0.301821285 |
| b-GWG | Lactobacillales | 0.576780302 | -0.068875304 |
| b-GWG | Firmicutes | 0.221362044 | -0.150242136 |
| a-GWG | Bacilli | 0.04495878 | -0.24398223 |
| a-GWG | Bacteroidia | 0.876623563 | -0.019180813 |
| a-GWG | Coriobacteriia | 0.064178517 | -0.225737532 |
| a-GWG | Alcaligenaceae | 0.134669158 | -0.183266848 |
| a-GWG | Bacteroidaceae | 0.474059314 | -0.08828141 |
| a-GWG | Coriobacteriaceae | 0.064178517 | -0.225737532 |
| a-GWG | Enterococcaceae | 0.14381558 | -0.179144445 |
| a-GWG | Prevotellaceae | 0.870468922 | -0.020145777 |
| a-GWG | Ruminococcaceae | 0.405655087 | -0.102475975 |
| a-GWG | Veillonellaceae | 0.565722949 | -0.070877982 |
| a-GWG | Bacteroides | 0.474059314 | -0.08828141 |
| a-GWG | Clostridium | 0.002257325 | -0.36430171 |
| a-GWG | Collinsella | 0.065228017 | -0.22487668 |
| a-GWG | Enterococcus | 0.1525228 | -0.175398732 |
| a-GWG | Megamonas | 0.488980282 | -0.085337925 |
| a-GWG | Prevotella | 0.669514081 | -0.052699977 |
| a-GWG | Ruminococcus | 0.74109623 | -0.040806988 |
| a-GWG | Bacteroidales | 0.876623563 | -0.019180813 |
| a-GWG | Coriobacteriales | 0.064178517 | -0.225737532 |
| a-GWG | Lactobacillales | 0.038506681 | -0.251566674 |
| a-GWG | Firmicutes | 0.159776218 | -0.172398061 |

**
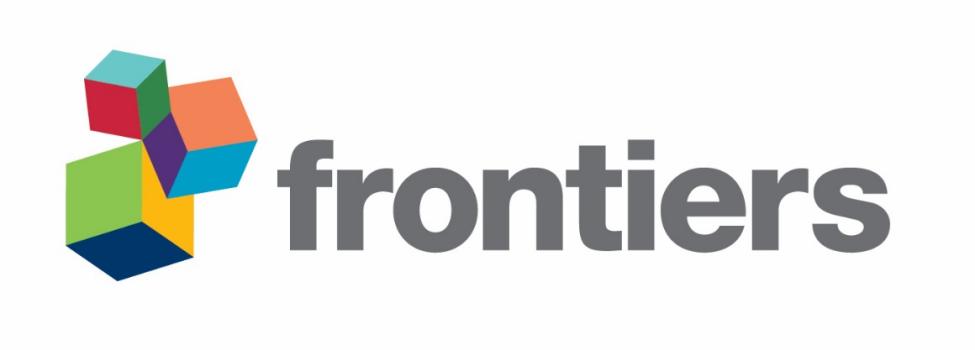
**
